# Construction of a bivalent vaccine against anthrax and smallpox using the attenuated vaccinia virus KVAC103

**DOI:** 10.1186/s12866-021-02121-5

**Published:** 2021-03-08

**Authors:** Deok Bum Park, Bo-Eun Ahn, Hosun Son, Young-Ran Lee, Yu-Ri Kim, Su Kyoung Jo, Jeong-Hoon Chun, Jae-Yon Yu, Myung-Min Choi, Gi-eun Rhie

**Affiliations:** 1 Division of High-risk Pathogens, Bureau of Infectious Disease Diagnosis Control, Korea Disease Control and Prevention Agency, Cheongju, South Korea; 2 Present address: Forensic DNA Division, Gwangju Institute, National Forensic Service, Jeonnam, South Korea; 3 Division of Vaccine Research, Korea National Institute of Health, Korea Centers for Disease Control and Prevention, Cheongju, South Korea; 4 Present address: Convergence Bioceramic Materials Center, Korea Institute of Ceramic Engineering and Technology, Cheongju, South Korea

**Keywords:** Anthrax, Smallpox, Vaccinia virus, IL-15, Cholera toxin

## Abstract

**Background:**

Anthrax and smallpox are high-risk infectious diseases, and considered as potential agents for bioterrorism. To develop an effective countermeasure for these diseases, we constructed a bivalent vaccine against both anthrax and smallpox by integrating a gene encoding protective antigen (PA) of *Bacillus anthracis* to the genome of the attenuated vaccinia virus strain, KVAC103.

**Results:**

Immunization with this bivalent vaccine induced antibodies against both PA and vaccinia virus in a mouse model. We also observed that the efficacy of this vaccine can be enhanced by combined immunization with immunoadjuvant-expressing KVAC103. Mouse groups co-immunized with PA-expressing KVAC103 and either interleukin-15 (IL-15) or cholera toxin subunit A (CTA1)-expressing KVAC103 showed increased anti-PA IgG titer and survival rate against *B. anthracis* spore challenge compared to the group immunized with PA-expressing KVAC103 alone.

**Conclusions:**

We demonstrated that the attenuated smallpox vaccine KVAC103 is an available platform for a multivalent vaccine and co-immunization of immunoadjuvants can improve vaccine performance.

**Supplementary Information:**

The online version contains supplementary material available at 10.1186/s12866-021-02121-5.

## Background

*Bacillus anthracis* and *Variola* virus are causative agents of anthrax and smallpox, respectively, and representative pathogens that can be possibly utilized as bioterrorism or biological weapons. Development of effective medical countermeasures against these pathogens is a national task of high priority [1, 2].

The biological attack in 2001 by *B. anthracis* spores via the US postal system has prompted the need to develop vaccines and therapeutics against anthrax [1]. Protective antigen (PA) is one of the major component of anthrax toxin, and also a principal ingredient of two licensed anthrax vaccines, Anthrax Vaccine Adsorbed (AVA) and Anthrax Vaccine Precipitated (AVP) [3]. Recently, a recombinant PA protein vaccine is being developed by Korea Centers for Disease Control (KCDC), and clinical trials are in progress [4, 5].

Although endemic smallpox was declared eradicated since the last case observed in 1977, *Variolar* virus still remains a potential biological weapon [2], and smallpox vaccines have been stockpiled for strategic use in some nations. To reduce side effects of conventional smallpox vaccines, attenuated vaccinia virus strains have been investigated in various ways [6]. KVAC103 is an attenuated vaccinia virus developed by KCDC [7].

Interleukin-15 (IL-15) is a cytokine involved in the proliferation and maintenance of CD8^+^ memory T cells, and has been suggested as an effective vaccine adjuvant [8, 9]. Previous studies on HIV-1 vaccine demonstrated that co-immunization of IL-15 strongly increased antigen-specific memory T cells and long-term immunity [10, 11]. Smallpox vaccines with integrated IL-15, tested in a mouse model, showed increased and prolonged cellular and humoral immunity [12]. This IL-15-containing smallpox vaccine also has been applied in a multivalent influenza vaccine [13]. Co-administration of IL-15 with staphylococcal enterotoxin B vaccine increased the number of dendritic cells in a mouse model [14].

Cholera toxin (CT) also has long been investigated as an efficient immunoadjuvant. The toxin is composed of subunit A and B, and subunit A contains two fragments, A1 and A2 [15]. The ADP-ribosyltransferase activity of cholera toxin subunit A1 (CTA1) is known to be important for enhancing immune responses [16]. The effect of CTA1 as an immunoadjuvant has been demonstrated against numerous pathogens, such as influenza A virus, HIV, *Helicobacter pylori*, and *Mycobacterium tuberculosis* [17–20].

Vaccinia virus is a popular platform for gene transfer and multivalent vaccine against various diseases [21, 22]. In a previous study, a dual vaccine for smallpox and anthrax has been developed by inserting PA gene of *B. anthracis* into Wyeth or modified vaccinia Ankara (MVA) strain [23]. A viral vector system that utilizes KVAC103 as a gene delivery system and a multivalent vaccine has been previously invented [7, 24]. In this study, we constructed a bivalent vaccine candidate against both smallpox and anthrax, by integrating a recombinant anthrax PA-encoding gene into KVAC103, using a viral vector pVVT1-EGFP-C7L. We examined the protective efficacy of KVAC103-derived bivalent vaccine in a mouse model. In addition, we observed that the vaccine supplemented with immunoadjuvant-expressing vaccinia viruses can increase immune response against anthrax.

## Results

**Fig. 1:**
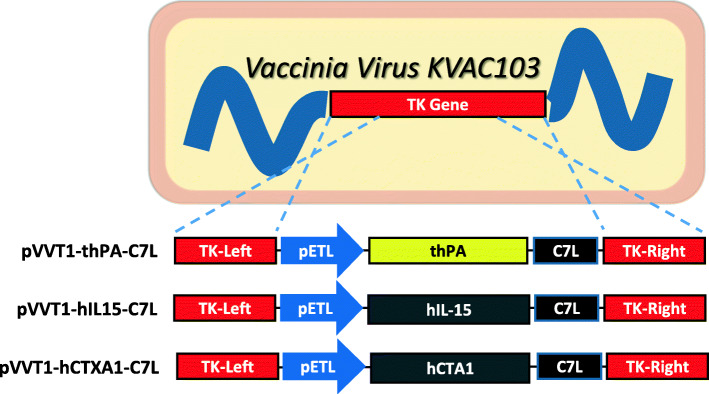
A diagram of viral vector construction. Human codon-optimized genes encoding PA with a signal peptide (MDAMKRGLCCVLLLCGAVFVSP) derived from the tissue plasminogen activator polypeptide (thPA), IL-15 (hIL15), or CTA1 (hCTA1) were cloned into pVVT1-EGFP-C7L. The GeneBank sequence ID for PA, IL-15, and CTA1 are AAA22637.1, NP_000576.1, and CAA24995.1, respectively. The expected molecular weight of integrated thPA, IL-15, and CTA1 are 81 kDa (757 aa), 18 kDa (162 aa), and 29 kDa (258 aa), respectively. Viral vector constructs are integrated into KVAC103 genome by homologous recombination at TK gene site

A human codon-optimized PA was cloned into viral vector pVVT1 to generate smallpox/anthrax dual vaccine candidate. A signal peptide derived from the tissue plasminogen activator was attached to the N-terminal of PA (thPA). We also constructed viral vector clones encoding a human IL-15 (hIL15) or a human codon-optimized CTA1 (hCTA1) gene. The viral vectors were integrated into the KVAC103 genome by homologous recombination at the thymidine kinase (TK) gene site (Fig. 1).

**Fig. 2:**
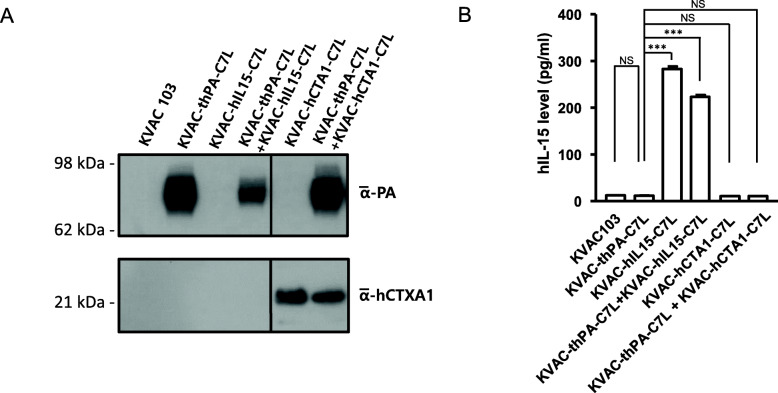
Expression of integrated proteins in vitro*.* **a** The expression of PA and CTA1 was detected by immunoblot assay in cell lysates. Vero cells were infected with KVAC-thPA-C7L and KVAC-CTA1-C7L for 48 h, and 50 μg of cell lysates from the infected cells were analyzed. The molecular weights from a size marker are indicated on the left. **b** The expression level of IL-15 was detected by ELISA. The IL-15 expression levels of infected Vero cells with KVAC103 derivatives were determined by the IL-15 ELISA kit (Biolegend). The bars on the graph indicates means of IL-15 expression levels from duplicated results in the same experiments, and the error bars stand for the standard error of the mean (***P < 0.001). NS, not significant. One-way ANOVA was applied for analysis

Protein expression of PA and CTA1 in the dual vaccine candidate viruses were confirmed by immunoblot assay (Fig. 2)a. PA and CTA1 were detected in virus-infected cell lysates. This indicates that cells infected by KVAC-thPA-C7L or KVAC-hCTA1-C7L viruses properly express PA or CTA1, respectively. The IL-15 ELISA result shows that cells infected by KVAC-hIL15-C7L also secreted IL-15 in vitro (Fig. 2b).

**Fig. 3:**
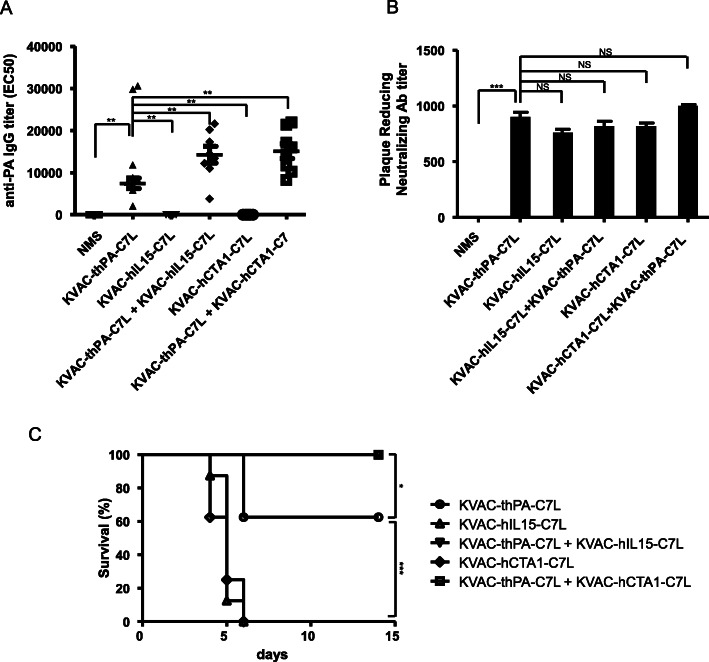
Immunogenicity and protective efficacy of the bivalent vaccine with or without adjuvant-expressing viruses in a mouse model. **a** Anti-PA IgG titers of individual mice in 5 groups (*n* = 8 for each group) were determined by ELISA. The Y-axis represents EC_50_ values. The horizontal bars indicate mean of individual groups (for KVAC-thPA-C7L, the mean value calculated except the two outliers). The error bars represent standard error of the mean. The asterisks (**) represent significant differences between indicated groups in statistical analysis (**P < 0.01). NMS, normal mouse sera. **b** Anti-viral antibody titers were determined by PRNT assay. The Y-axis represents PRNT_50_, the reciprocal of the dilution factor of sera reducing plaque formation in half. The bars represent arithmetic means of results from two independent assays with pooled sera of individual groups (eight mice per group) and the error bars represent standard error of the mean (***P < 0.001). NS, not significant. NMS, normal mouse sera. **c** Immunized mice were challenged with 50 × LD_50_ of *B. anthracis* Sterne spores by *s.c.* injections. Survival rates of 5 groups (*n* = 8 for each group) were observed for 14 days. The *p*-value between KVAC-thPA-C7L and KVAC-C7L with immunoadjuvant only (KVAC-hIL-C7L, KVAC-hCTA1-C7L) is lower than 0.001 and indicated as ***. The *p*-value between KVAC-thPA-C7L and both KVAC-C7L with PA and immunoadjuvant (KVAC-thPA-C7L + KVAC-hIL-C7L, KVAC-thPA-C7L + KVAC-hCTA1-C7L) is lower than 0.05 and indicated as *

In a preliminary experiment, we observed that repeated vaccination in 3 week interval increased the anti-PA antibody titer around 10-fold compared to single vaccination (data not shown). The in vivo efficacy of the dual vaccine candidate with or without adjuvant-expressing viruses was estimated in a mouse model (Fig. 3). We immunized A/J mice (*n = 8*) with our vaccine candidate KVAC-thPA-C7L with or without adjuvant expressing viruses 2 times with a 3-week interval. The anti-PA antibody levels of all groups immunized with KVAC-thPA-C7L were increased compared to the groups immunized with the adjuvant only (KVAC-hIL15-C7L or KVAC-hCTA1-C7L). Mouse groups vaccinated with KVAC-thPA-C7L plus an immunoadjuvant-containing strain (KVAC-hIL15-C7L or KVAC-CTA1-C7L) exhibited higher mean values of antibody titers compared to the group immunized with KVAC-thPA-C7L only (Fig. 3a). Except the two outliers which are extraordinarily high in the group immunized with KVAC-thPA-C7L only in Fig. 3a (29,800 and 30,600), the mean values of anti-PA antibody titer are significantly increased in the mouse groups immunized with both KVAC-tPA-C7L and adjuvant-expressing strains (One-way ANOVA, *p* value < 0.01).

Neutralizing antibodies against vaccinia virus in mouse sera were measured by PRNT assay. Unlike the anti-PA antibodies, production of neutralizing antibodies against vaccinia virus does not appear to be significantly affected by the presence of immunoadjuvant (Fig. 3b). In a previous study, IL-15 expressing vaccinia virus induced increased neutralizing antibodies compared to the control vaccinia virus in a mouse model [12]. In our experiment, the effect of adjuvants was not observed and all the sera immunized with the constructs induced similar level of neutralizing antibodies.

Immunized mice were challenged with *B. anthracis* Sterne spores 3 weeks after the final vaccination. Survival rates were monitored for 2 weeks. Mice immunized with adjuvant only were all dead within a week. In the group immunized with KVAC-thPA-C7L only, 62.5% of mice survived, while groups immunized with both KVAC-thPA-C7L and immunoadjuvant expressing virus (KVAC-hIL15-C7L or KVAC-CTA1-C7L) were fully protected from the challenge (Fig. 3c). The result indicates that enhanced immunity achieved by co-expression of adjuvant can protect the mice more effectively against anthrax.

## Discussion

Poxviruses have been often used as a vector system for vaccines because of their large DNA genome and convenience in manipulation [21, 22]. In a previous study, engineered vaccinia strains expressing both PA and IL-15 showed enhanced immunogenicity against *B. anthracis* compared to the conventional anthrax vaccine AVA in animal test [23]. Our result presented that the co-expression of IL-15 in KVAC103 also enhanced protective efficacy of our bivalent vaccine. Co-expression of CTA1 induced immune response against the PA-expressing vaccine in the similar level to IL-15. Our result demonstrated that co-immunization of CTA1, as well as IL-15, was effective enough to enhance the immune responses against PA and reconfirmed that CTA1 is a suitable adjuvant for multivalent vaccines derived from KVAC103. This result is the first observation of the effect of CTA1 as an immunoadjuvant in a viral vaccine system.

## Conclusion

In summary, we explored the possibility of developing a bivalent vaccine using KVAC103, an attenuated vaccinia virus strain. Like other vaccinia virus strains previously utilized, it is confirmed that KVAC103 also can serve as a useful platform for multivalent vaccines. In addition, the vaccine can be further effective with the supplement of cytokines or adjuvants.

## Methods

### Cell and virus

Vero cell (African green monkey kidney cell) was purchased from the American Type Culture Collection (ATCC, USA). Cells were grown in Opti-Eagle’s Minimum Essential Medium (Opti-MEM, Invitrogen) supplemented with 2% heat-inactivated fetal bovine serum (FBS, Invitrogen), incubated at 37 °C, and humidified with 5% CO_2_. The attenuated vaccinia virus strain KVAC103 and the viral vector pVVT1-EGFP-C7L were provided by Korea National Institute of Health (KNIH). This vector contains the vaccine virus C7L gene which encodes interferon antagonist, and this is one of the 26 genes defective in KVAC103 compared to its ancestor strain. This gene is required for enhanced viral reproduction [24].

### Construction of anthrax/smallpox dual vaccine candidate vectors

Viral vector constructs were generated using the pVVT1-EGFP-C7L vector [24] as a template (Fig. 1). Human IL-15 gene and human codon-optimized *B. anthracis* PA and CTA1 genes were synthesized (Bioneer). The synthesized genes were cloned into the vector using *Sfi*I restriction enzyme site. The constructed vectors were mixed with Lipofectamine (Invitrogen) and transfected into KVAC103-infected Vero cells. Single plaques were isolated from the original infected cells and verified using PCR. The primer sequences used for verification were 5′-TTT GAA GCA TTG GAA GCA ACT-3′ and ‘5’-ACG TTG AAA TGT CCC ATC GAG T-3′.

### Virus preparation

Viruses were infected to mono-layered Vero cells with 0.01 MOI. The virus-infected cell media were harvested when more than 80% of total cells showed cytopathic effect. From the harvested culture supernatant, viruses were collected by ultra-centrifugation. The pellet was resuspended in 1× PBS, pH 7.0 (Gibco). The concentration of viral particles was determined by the standard plaque assay. The viruses were infected the Vero cells overlaid on 6-well plates for 2 days. The plate were staining with crystal violet and the plaque numbers on each well were counted.

### Western blot analysis

Virus-infected Vero cells or their culture supernatants were lysed in 1× RIPA buffer (G-Bioscience) containing 1% PMSF (Theromfisher Scientific) at 4 °C. Fifty μg of protein from each cell lysate was resolved on denaturing polyacrylamide gel electrophoresis (PAGE) and transferred to polyvinylidene difluoride (PVDF) membrane (Amersham). Expression levels of PA and CTA1 proteins were detected using mouse monoclonal antibodies against PA and cholera toxin, respectively (Abcam, 1:1000), and horse radish peroxide (HRP)-conjugated secondary antibodies (Abcam, 1:3000).

### Mouse immunization and serum collection

**Fig. 4:**
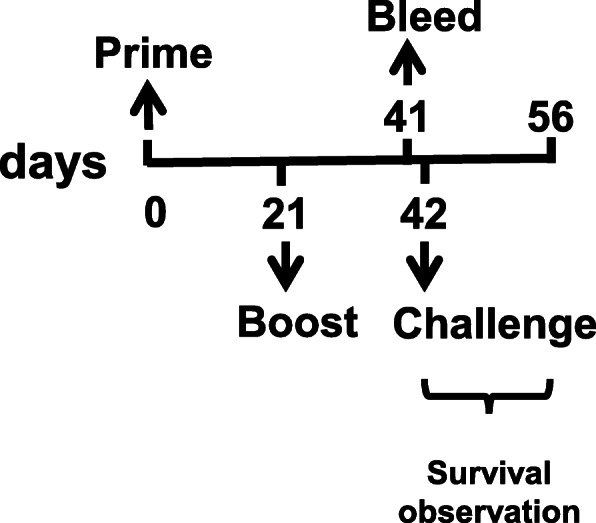
A Schematic view of the animal experiment. Mice were immunized two times with 3 weeks interval and sera were collected by bleeding 20 days after the last vaccination. One day after bleeding, mice were challenged with anthrax spores. Survival was observed for 14 days

Female A/J mice (5-week old) were purchased from SLC, Inc. (Japan) and housed in an animal biosafety level 2 (ABL2) facility in KCDC. Mice were immunized with the vaccine candidate virus (5 × 10^7^ pfu/mouse) with or without the adjuvant-expressing virus (5 × 10^7^ pfu/mouse) 2-times at 3-week intervals subcutaneously (*s.c.*) with 8 mice as a group. Mice sera were collected 20 days after final immunization to measure anti-PA IgG and vaccinia virus plaque reduction neutralizing antibody titers. The schematic view of mouse immunization and serum collection is in Fig. 4.

### Enzyme-linked immunosorbent assay (ELISA)

The anti-PA IgG titers of mice sera were determined by ELISA as previously described with some modifications [25]. Briefly, 96 well plates were coated with 1 μg/ml of recombinant PA (Green Cross, Korea). Mouse sera were diluted from 1:100 to 1:204800 and loaded to each well, incubated for 1 h at 37 °C. Horseradish peroxidase-conjugated anti-mouse IgG goat antibody (Invitrogen) and 3,3`,5,5`-tetramethylbenzidine (TMB) substrate were used for detection. The optical density of each well was measured at 450 nm and the half maximal effective concentration (EC_50_) was calculated by 4-parameter logistic equation regression using SoftMaxPro5.3 (Molecular Device, USA). The data were analyzed and visualized using GraphPad Prism 5.

The IL-15 expression level of KVAC103 derivatives were determined by the IL-15 ELISA kit (Biolegend) according to the manufacturer’s protocol. Vero cells were infected with viruses of 0.01 MOI. Cell lysates were collected 2 days after infection and analyzed.

### Plaque reduction neutralization test (PRNT)

Serial two-fold dilutions of heat-inactivated mouse sera were mixed with vaccinia virus Lister strain of approximately 50 plaque forming units (PFU). After 2 h incubation at 37 °C, the serum and virus mixtures were inoculated onto monolayered Vero cells. After two days incubation at 37 °C with 5% CO_2_, cells were fixed and stained using a mixture of crystal violet and formalin for 10 min. Stained plates were dried in air at room temperature and the plaque numbers were counted. The neutralizing antibody titer was defined as the reciprocal of dilution factor that reduced plaque numbers in half (50%) compared to a serum-free control (PRNT_50_).

### *B. anthracis* spore challenge

Immunized mice were challenged with 50-fold of lethal dose 50 (LD_50_) of *B. anthracis* Sterne spore by *s.c.* injections. Survival of the mice was monitored for 14 days as described in Fig. 4. Spores were prepared according to a previous study [26]. The LD_50_ determined by Reed-Muench method [27] in A/J mice model via *s.c.* route was 1794 spores. Survived animals were euthanized using CO_2_ gas. Animal study protocols (KCDC-102-16-2A and KCDC-039-17-2A) were approved by the Institutional Animal Care and Use Committee (IACUC) of Korea Centers for Disease Control and Prevention (KCDC). All procedures involved in the housing and care of animal strictly followed guidelines and requirements of the IACUC.

### Statistical analysis

The Statistical analysis was performed using GraphPad Prism 5. To analyze the anti-PA ELISA titer, One-way ANOVA followed by Tukey’s post hoc test were used to evaluate the difference between groups. To analyze the survival rate, Kaplan-Meier survival plots were evaluated with the log-rank test.

## Supplementary Information

**Additional file 1.**

**Additional file 2.**

**Additional file 3.**

## Data Availability

The datasets used and/or analyzed during the current study are available from the corresponding author on reasonable request.
