# Supplementary material for: Construction of a bivalent vaccine against anthrax and smallpox using the attenuated vaccinia virus KVAC103
Source: BMC Microbiol. 2021 Mar 8;21:76. doi: 10.1186/s12866-021-02121-5 (PMC7938549; doi:10.1186/s12866-021-02121-5)
Supplement: Supplementary file 1 — Additional file 1. [file 12866_2021_2121_MOESM1_ESM.docx]

Additional file 1: Original full-length blot of Fig. 2A

1 2 3 4 5 6 7 8


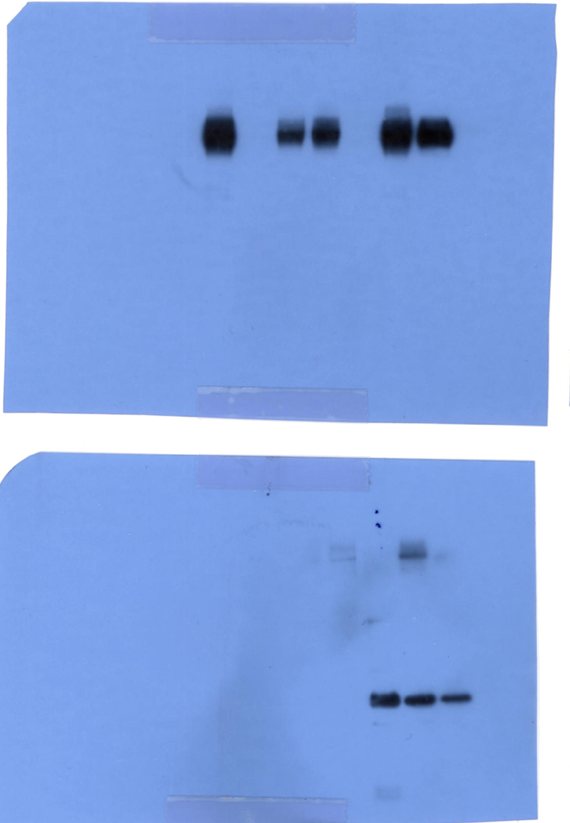


1. KVAC103
2. KVAC-thPA-C7L
3. KVAC-hIL15-C7L
4. KVAC-thPA-C7L + KVAC-hIL15-C7L
5. KVAC-hIL15-thPA-C7L
6. KVAC-hCTXA1-C7L
7. KVAC-thPA-C7L + KVAC-hCTXA1-C7L
8. KVAC-hCTXA1-thPA-C7L

We have also constructed PA-immunoadjuvant co-integrated vaccine candidates (lane 5 and 8), and confirmed them by western blot. However, we didn’t include the result in this manuscript because the results of other experiments with these two vaccine candidates seemed unstable. The image of Fig.2A in the ‘Figure’ file was replaced according to the editorial policy.
