# Supplementary material for: Construction of a bivalent vaccine against anthrax and smallpox using the attenuated vaccinia virus KVAC103
Source: BMC Microbiol. 2021 Mar 8;21:76. doi: 10.1186/s12866-021-02121-5 (PMC7938549; doi:10.1186/s12866-021-02121-5)
Supplement: Supplementary file 3 — Additional file 3. [file 12866_2021_2121_MOESM3_ESM.docx]

As a preliminary experiment, we vaccinated mice 1 time only or 2 times with a 3 week interval and observed the induction of anti-PA IgG titers. As seen in the Fig. below, two vaccinations significantly increased the anti-PA IgG titers at 6 weeks compared to the one injection of vaccine. This result indicates that the first vaccination did not negatively affect the second vaccination for PA part. As for the vaccinia virus part, we did not measure anti-viral antibody titers after the first vaccination.


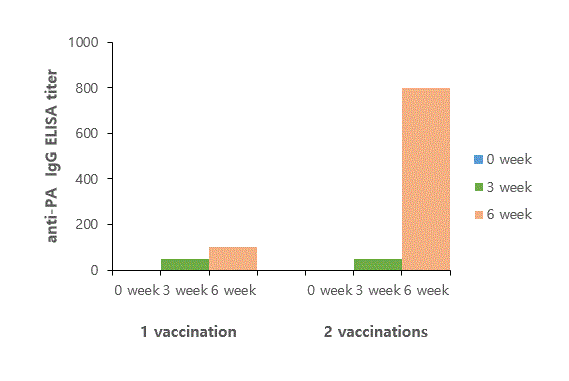


This experiment was performed with pooled sera from each mouse group (n=5) in 2013. The method for anti-PA IgG ELISA was different to the one used in this paper as well as the calculation method for antibody titers. The endpoint titers for this experiment were defined as the reciprocal of the highest serum dilution that resulted in an absorbance three standard deviations greater than the average absorbance of negative control serum samples at the same dilution (Rhie et al. FEMS Immunol Med Microbiol (2005), 45: 331-339). As the result is preliminary without repetition, we would not like to present this result in the paper. In addition, in the Perera paper (PNAS (2010) 107(42): 18091-18096), no negative effects were observed either on PA or vaccinia virus parts.
